# Issue of Veracity between Dr. Robert Battey and Mr. Lawson Tait

**Published:** 1886-06

**Authors:** 


### ISSUE OF VERACITY BETWEEN DR. ROBERT BATTEY AND MR. LAWSON TAIT.

The following letter from Dr. Robert Battey, in the *Medical News*, May 15th, touching an error in the statement of results of Battey's and Tait's operation, is calculated to impress upon American readers certain misgivings as to the trustworthiness of the reports made by Mr. Lawson Tait in regard to his gynecological work. If an operator varies his statement of results in given cases to suit his ulterior ends or to meet the issues presented by the experience of others, we have no safe guide to a discussion of any mooted question in the published data of such surgeon, and Mr. Tait should know that "facts are stubborn things:"

In his "Note on Removal of the Uterine Appendages" (page 456, current volume of the *Medical News*), Mr. Lawson Tait inadvertently says of my original operation: "It was first performed by Battey, with a *fatal* result, on August 17, 1872, and therefore, if proper names are to be given to the operations, it deserves to have Dr. Battey's name attached to it."

This case has been so often reported to medical societies, and cited in the journals, in London as well as in America, as a *successful* case, that I can hardly suppose Mr. Tait to be ignorant of the fact. I therefore accept it as a slip of the pen in the hurry of a busy professional life. I can the more readily do this since, in the same connection, he is quite as unhappy in reporting the result of his own case, when he tells us "it was first performed by me on August 1, 1872, with a *successful* result. I am therefore entitled to have this operation described by my name."

In the *British Medical Journal* for May 31, 1879, we find from the pen of Mr. Tait the following: "Removal of Normal Ovaries. As a small contribution to the history of this proceeding, I should like to supplement Professor Simpson's paper by the statement that I have removed the ovaries for the arrest of hemorrhage in cases of myoma three times, *in all three with a fatal result.* The dates were August 1, 1872; December 26, 1873, and March 14, 1874. It will thus be seen that this operation was performed in England five days *after* it was first performed in Germany, and sixteen days *before* it was performed by Dr. Battey. . . . That this operation will prove a great addition to surgery I have no doubt. With our improved methods of operation, I believe that at least two, possibly all three, of my cases would recover now if I had them over again."

To Mr. Tait's claims of priority I have nothing to say. For more than six years (from September, 1872, to May, 1879), during which this subject was actively discussed in medical societies and medical journals, his voice was not heard. There seems little disposition manifested anywhere to re-open the case now for his benefit.

### ITEMS.

DR. K. H. DAVIS, of Cole City, Georgia, was in Atlanta a few days since and gave THE JOURNAL a pleasant call.

WE had a pleasant call a few days since from Chas. P. Frick, the representative of the well-known drug-house of R. A. Robinson & Co., of Louisville, Kentucky.

DR. W. F. WESTMORELAND, the senior editor of THE JOURNAL, has been quite sick recently. We are pleased to state that he is improving and will soon be well again.

WE will publish in the July number of THE JOURNAL an able article from Dr. J. P. Logan, of this city, on the "Relation of the Medical Profession to the Use and Abuse of Alcoholic Liquors."

THERE are only two members of the Medical Association of Georgia living who were present at the organization of the Association in 1849—Dr. S. D. Brantley, of Sandersville, and Dr. James F. Alexander, of this city.

IN the May number of THE JOURNAL there appeared an error in the advertisement of Dr. A. G. Hobbs. The card appears corrected in this number.

WE have received the prospectus of *Progress*, a new journal which will be published in Louisville by D. W. Raymond, and edited by Dr. Dudley S. Reynolds. The first number will be issued July 1st.

DR. A. B. ASHWORTH, of this city, who has been in the office of THE JOURNAL for the last two years, has been very dangerously sick with pneumonia for several weeks. He is improving now, and we hope to see him out in a short time.

DR. R. C. STOCKTON REED, Dean of the Cincinnati College of Medicine and Surgery, was in Atlanta in May. He called at our office and we were out. We regret exceedingly we did not meet him, and hope that he will come this way again.

DR. C. W. NUTTING, of Etna, California, a former citizen of this city, after spending the winter in the New York Polyclinic, passed through Atlanta on his way home a few days ago. The doctor was at one time Demonstrator of Anatomy in the Atlanta Medical College.

OFFICERS OF THE AMERICAN MEDICAL ASSOCIATION.—*President*—E. H. Gregory, St. Louis, Missouri. *Vice-Presidents*—*First*, B. H. Miller, Stillwater, Minn.; *Second*, W. B. Welch, Arkansas; *Third*, Wm. H. Pancoast, Philadelphia; *Fourth*, William C. Wile, Connecticut. *Permanent Secretary*—William B. Atkinson, Philadelphia. *Assistant Secretary*—J. Nevins Hyde, Chicago. *Treasurer*—Richard J. Dunglison, Philadelphia. *Librarian*—C. H. A. Kleinschmidt, Washington, D. C. *Committee on Necrology*—J. M. Toner, Washington, D. C., Chairman. *Judicial Council*—N. S. Davis, Illinois; H. Brown, Kentucky; William Brodie, Michigan; D. J. Roberts, Tennessee; R. C. Moore, Nebraska;

T. A. Foster, Maine; James A. Gray, Georgia. *Trustees of the Journal of the American Medical Association*—P. O. Hooper, Arkansas; Alonzo Garcelon, Maine; L. S. McMurtry, Kentucky. The next meeting will be held in Chicago, beginning the first Tuesday in June, 1887.

TRIBUTE OF RESPECT.—At a meeting of the physicians of this city held, May 19th, the following resolutions were adopted :

WHEREAS, By the providence of God, death has removed from our midst our distinguished brother, Dr. John M. Johnson, for so many years a leader in his profession, and by his wide knowledge a guide to his brethren and a benefactor to humanity; therefore be it

*Resolved*, That we, members of the medical profession of Atlanta, assembled to do honor to his memory, do recognize in his death that a great and good man has gone to his just reward, and that in his long and blameless life we find an example worthy of our emulation and reverence.

*Resolved*, That we extend to his family and relatives our warm sympathy in this their great bereavement, believing at the same time that their sorrow must be tempered by the realization of the fact that his well-rounded life reached its rich fruition, and left behind him many hearts to mourn his loss and remember his kindly and charitable ministrations.

*Resolved*, That the medical profession, as a mark of respect, attend his funeral in a body.

*Resolved*, That these resolutions be published in the daily papers and in the *Southern Medical Record* and THE ATLANTA MEDICAL AND SURGICAL JOURNAL.

WM. PERRIN NICOLSON, M. D.,
E. L. CONNALLY, M. D.,
J. G. EARNEST, M. D.,
J. C. AVARY, M. D.,
N. O. HARRIS, M. D.,
JOHN C. OLMSTED, M. D.,
Committee.

ONE of the most interesting features of THE JOURNAL is the lectures of Dr. Henry Wile, Lecturer on Dermatology in the Atlanta Medical College, which appear regularly every month. The one presented in this issue on general dermato-pathology is especially interesting.

THE circulation of THE JOURNAL is growing more rapidly now than ever before. From March 1st of this year to May 25th, we have received 811 subscribers, distributed as follows: Georgia, 365; Alabama, 127; South Carolina, 48; North Carolina, 54; Tennessee, 15; Florida, 21; Texas, 45; Louisiana, 57; Arkansas, 30; Mississippi, 27; New York, 12; Missouri, 3; Virginia, 5; District of Columbia, 2. This rapid increase shows the high estimation placed on THE JOURNAL by the profession everywhere.

YELLOW FEVER INOCULATION.—Dr. J. McF. Gaston, of this city, presented a preamble and resolution touching yellow-fever inoculation, at the recent meeting of the American Medical Association, which received the sanction of the Association, and a committee was authorized to memorialize Congress in favor of the pending bill to appoint a commission and appropriate funds for the investigation of this prophylactic measure.

METEOROLOGICAL SUMMARY FOR APRIL, 1886, STATION, ATLANTA, GA.

| | |
|---|---|
| Mean Temperature, | 60.4 |
| Highest Temperature, April 23rd, | 80.0 |
| Lowest Temperature, April 8th, | 32.0 |
| Monthly Range of Temperature, | 51.0 |
| Greatest Daily Range of Temperature, | 32.0 |
| Least Daily Range of Temperature, | 6.0 |
| Mean Daily Range of Temperature, | 18.0 |
| Prevailing Direction of Wind, | S. E. |
| Total Movement of Wind, | 7,862 Miles. |
| Highest Velocity of Wind and Direction, | 33 Miles, N. W. |
| Total Precipitation. | 25.2 inches. |
| Dates of Frosts { Light, | — |
| Dates of Frosts { Killing, | 1, 6, 8 |
| Dates of Thunder-storms, | 20, 21, 28, 29 |

